# Infections Associated with Medtronic Duet External Ventricular Drains — Rhode Island Hospital, Providence, Rhode Island, January 2023–January 2024

**DOI:** 10.15585/mmwr.mm7314a4

**Published:** 2024-04-11

**Authors:** Kevin M. Gibas, Dianne Auld, Stephanie Parente, Jean Horoho, Leonard A. Mermel

**Affiliations:** ^1^Department of Epidemiology and Infection Prevention, Rhode Island Hospital, Providence, Rhode Island; ^2^Department of Medicine, The Warren Alpert Medical School of Brown University, Providence, Rhode Island.

**Summary**

**What is already known about this topic?**

External ventricular drain (EVD) insertion is a common neurosurgical procedure. Disconnections and breaks of EVD catheters or connecting drainage system components result in cerebrospinal fluid (CSF) leakage and increased risk for EVD-associated infections.

**What is added by this report?**

Investigation of a hospital cluster of positive CSF cultures and EVD-associated infections identified frequent disconnections and breaks of Medtronic Duet EVD systems in September 2023, after a change to this system from the system previously used.

**What are the implications for public health practice?**

The Medtronic Duet product was recalled in January 2024. This investigation highlights the importance of hospital infection prevention and control programs in identifying, responding to, and preventing health care–associated infections.

## Abstract

External ventricular drains (EVDs) are medical devices that are inserted into the ventricles of the brain to drain excess fluid, manage intracranial hypertension, monitor intracranial pressure, and administer medications. Unintentional disconnections and breaks or fractures (breaks) of EVDs or associated drainage system components can result in cerebrospinal fluid (CSF) leakage and increased risk for EVD-associated infections. After replacement of Integra Life Sciences EVD systems with Medtronic Duet EVD systems at Rhode Island Hospital in mid-September 2023, a threefold increase was observed in the prevalence of positive CSF cultures, from 2.8 per 1,000 days with an EVD in place (EVD days) during January–September 2023 to 11.4 per 1,000 EVD days during October 2023–January 2024 (rate ratio [RR] = 5.7; 95% CI = 1.5–22.0; p = 0.01) and an eightfold increase in the prevalence of infections, from 0.7 to 6.5 per 1,000 EVD days (RR = 9.8; 95% CI = 1.1–87.3; p = 0.04). An investigation by Rhode Island Hospital Infection Control during December 2023–January 2024 identified frequent reports of disconnections and breaks of the Medtronic Duet EVD system. A search of the Food and Drug Administration Manufacturer and User Facility Device Experience database identified 326 reports nationwide of disconnection and breaks of components of the Duet EVD system, including 175 during 2023. A Medical Product Safety Network report was filed. The Duet EVD product was ultimately recalled in January 2024, citing disconnections of the EVD system and reports of CSF leakage and infection. Given the widespread use of EVD systems by neurosurgery centers and the risk for EVD-associated infections, a strategy for future consideration by hospital infection prevention and control programs might be inclusion of EVD-associated infections in hospital surveillance programs to rapidly identify increases in these events and determine factors related to such infections to prevent additional infections.

## Introduction

External ventricular drains (EVDs) are devices placed into the ventricles of the brain to drain excess fluid (e.g., cerebral spinal fluid [CSF] and blood), manage intracranial hypertension, monitor intracranial pressure, and administer medications (*1*). An EVD system consists of multiple components including a drain (the EVD); connecting tubing, stopcocks, transducer, and monitor; leveling manifold; and a CSF collecting reservoir (*2*). Approximately 25,000 EVDs are placed annually, making EVD insertion among the most common and important lifesaving neurosurgical procedures performed in the United States (*3*). Unintentional disconnections and breaks or fractures (breaks) of the EVD or the connecting drainage system tubing can result in CSF leakage and contamination of the EVD system, increasing the risk for EVD-associated infections, including meningitis and ventriculitis (*4*,*5*).

Rhode Island Hospital, a large academic hospital in Providence, Rhode Island designated as a level 1 trauma center, performs surveillance for EVD-associated infections using the National Healthcare Safety Network (NHSN) definition of patients with infection (i.e., meningitis or ventriculitis) and EVDs in situ for >48 hours (*6*). CSF specimens are collected from patients with EVDs in place at the discretion of the neurosurgery or neurocritical care unit (NCCU) teams, most frequently when a patient is symptomatic or if providers have concerns about possible infection. All CSF specimens with organisms identified on Gram stain or culture (positive CSF culture) trigger an alert to the Infection Control Team, which determines if the case meets criteria for an EVD-associated infection. Although a positive CSF culture is the initial requirement to meet criteria for a confirmed EVD infection, a patient with a positive CSF culture would not meet criteria for a confirmed EVD-associated infection if all the following four conditions are present 1) a positive CSF culture with a common commensal organism, 2) no symptoms consistent with meningitis or ventriculitis, 3) determination by the clinical team that the patient does not clinically have meningitis or ventriculitis, and 4) the patient did not receive dedicated treatment for meningitis or ventriculitis. Alternatively, if the positive CSF culture could be attributed to a noncentral nervous system primary source infection, the patient would meet the criteria.

In mid-September 2023, the emergency department and NCCU at Rhode Island Hospital replaced the Integra Life Sciences EVD system with the Medtronic Duet EVD system because of limitations in availability of the Integra Life Sciences product; hospital operating rooms continued to use Integra Life Sciences EVD systems. This report describes investigation of a cluster of positive CSF cultures and confirmed EVD-associated infections identified at Rhode Island Hospital after the switch to the Medtronic Duet EVD system. The hospital’s Institutional Review Board has determined that outbreak investigations are nonresearch and fall under the authority of infection control.*

## Investigation and Results

During January–September 2023, over 1,498 days with an EVD in place (EVD days), four patients with EVDs, including one with a confirmed infection, had positive CSF cultures^†^ (2.8 positive cultures and 0.7 infections per 1,000 EVD days) (Table). During October 1, 2023–January 10, 2024, over 614 EVD days, seven patients had positive CSF cultures, including four with confirmed EVD infections (11.4 positive cultures and 6.5 infections per 1,000 EVD days), representing an approximate threefold increase in positive CSF cultures (rate ratio [RR] = 5.7; 95% CI = 1.5–22.0; p = 0.01) and an eightfold increase in the prevalence of infections (RR = 9.8; 95% CI = 1.1– 87.3; p = 0.04) after transition to the Medtronic Duet EVD system^§^ (*7*,*8*).

**TABLE:** Positive cerebrospinal fluid cultures and confirmed infections in patients with external ventricular drains — Rhode Island Hospital, Providence, Rhode Island, January 2023–January 2024*

Month/Year	No. of EVD events with positive CSF cultures	No. of events meeting NHSN infection criteria^†^	EVD days^§^	Positive CSF culture rate^¶^	EVD-associated infection rate**
**Total (Jan–Oct 2023)**	**3**	**1**	**1,498**	**2.8**	**0.7**
Jan 2023	0	0	238	—	—
Feb 2023	0	0	152	—	—
Mar 2023	1	0	204	4.9	—
Apr 2023	0	0	194	—	—
May 2023	1	0	169	5.9	—
Jun 2023	0	0	173	—	—
Jul 2023	0	0	121	—	—
Aug 2023	0	0	136	—	—
Sep 2023^††^	1	1	111	9.0	9.0
**Total (Oct 2023–Jan 2024)**	**7**	**4**	**614**	**11.4**	**6.5**
Oct 2023	0	0	131	—	—
Nov 2023	2	0	177	11.3	—
Dec 2023	4	3	201	19.9	14.9
Jan 2024*	1	1	105	8.8	8.8

**Abbreviations:** CSF = cerebrospinal fluid; ED = emergency department; EVD = external ventricular drain; NCCU = neurocritical care unit; NHSN = National Healthcare Safety Network.

* Through January 10, 2024.

^†^ EVD-associated infections were determined using the NHSN surveillance definition of infection (i.e., meningitis or ventriculitis) for patients with an EVD in situ for >48 hours.

^§^ EVD days are calculated as the sum of the total number of days an EVD is in place for each patient admitted to Rhode Island Hospital who received an EVD.

^¶^ Number of positive CSF cultures per 1,000 EVD days.

** Number of EVD infections per 1,000 EVD days.

^††^ Medtronic Duet EVD systems were introduced in NCCU and ED the week of September 11, 2023. The positive cultures and infections noted in September 2023 were confirmed to have occurred before the transition to the Medtronic Duet EVD systems.

CSF cultures of specimens obtained from seven patients during October 2023–January 2024 were positive for bacterial growth; five of these cultures grew coagulase-negative staphylococci, one grew *viridans group streptococci*, and another grew *Streptococcus gordonii*, *Streptococcus salivarius*, and *Rothia* species. Six of these seven patients had received Medtronic Duet EVD systems, and one had received an Integra Life Sciences EVD system. The patient who received the Integra Life Sciences EVD had their EVD placed in the operating room; all patients who received the Medtronic EVDs had them placed in either the emergency department or NCCU.

As part of the investigation, information was collected about the providers who cared for the patient (including their level of training) and hospital locations occupied by affected patients or where they were provided care. Specimen collection procedures, infection control practices, and the data from the hospital's adverse events reporting system (SafetyNet) were also reviewed.

### Investigation of Staff Members and Hospital Locations

No common health care providers (including surgeons, house officers, nursing staff members, and staff members who inserted the EVDs or collected EVD cultures) or geographic locations or units were shared by all seven patients. Further, no recent changes were identified in procedures for collecting or processing CSF specimens or in Rhode Island Hospital’s EVD infection prevention program.

Interviews with neurosurgeons and NCCU staff members revealed reports of frequent unintentional disconnections and breaks of the Medtronic Duet EVDs tubing, which had not been observed with the Integra Life Sciences EVD system that was previously used at Rhode Island Hospital. Staff members reported that these events consisted of the collecting tubing disconnecting from the patient-line stopcock connectors (Figure). When this issue was identified, nursing staff members, neurosurgeons, and NCCU providers were notified to report these events in SafetyNet, the Rhode Island Hospital adverse event reporting system, to track these occurrences. No issues with staff member training related to the device, improper use of the device by staff members, or any other staff-related issues were identified.

**FIGURE:**
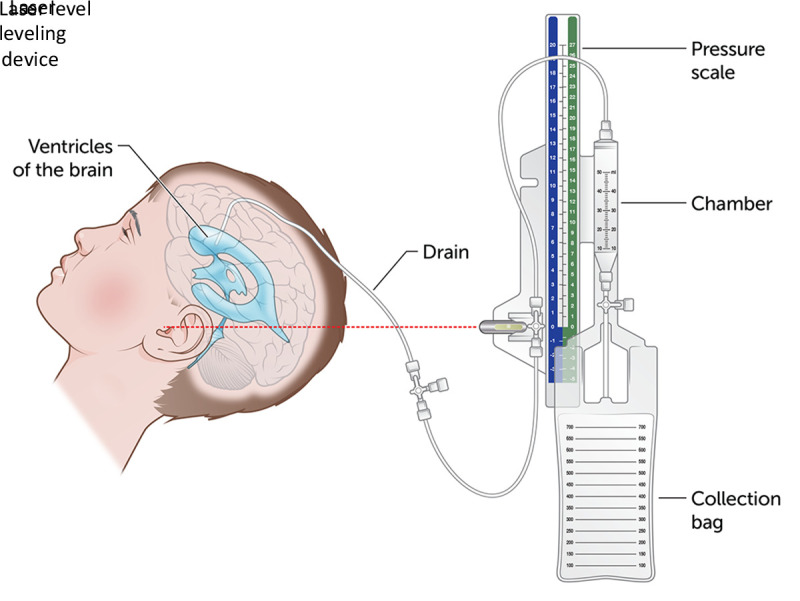
Components of an external ventricular drain system **Source:** The Royal Children’s Hospital, Melbourne, Australia; republished with permission.

### Review of SafetyNet Data

Review of the Rhode Island Hospital SafetyNet data found no reports of improper use of the Medtronic EVD system and no reports of adverse events related to EVD systems before the switch to the Medtronic Duet EVD system in September 2023; however, after transitioning to the Medtronic EVD system, nine EVD-related adverse events were reported in SafetyNet, all related to disconnections or breaks of Medtronic Duet EVDs. A search of the Food and Drug Administration (FDA) Manufacturer and User Facility Device Experience (MAUDE) database using the search term “Medtronic Duet” identified 326 medical device reports involving malfunction of the Medtronic Duet EVD system, including 175 (54%) reports during 2023, 120 (69%) of which were for disconnection of components of the EVD system, and 23 (13%) of which were breaks in the EVD system^¶^ (*9*).

## Public Health Response

Because of concern that the increase in observed positive CSF cultures and EVD-associated infections at Rhode Island Hospital could be related to disconnections and breaks of components in the Medtronic Duet EVD system, Rhode Island Hospital stopped placing new Medtronic Duet EVDs in patients in early January 2024 and transitioned to an alternative product. Patients who already had Medtronic EVDs in place at the time of these findings had their EVDs maintained until removal was clinically indicated or warranted. Rhode Island hospital also filed individual FDA Medical Product Safety Network reports for each of the confirmed EVD infections observed after transitioning to the Medtronic product on January 9, 12, 24, and 31, 2024. On January 24, 2024, Medtronic issued a voluntary recall of the Duet EVD system products, citing the potential for catheter disconnection from the patient-line stopcock connectors, and noting that disconnections at the stopcock connection in the affected Duet EVD system might occur at any point along the patient line or tubing (*10*) (Figure). Information in the recall notice indicated that cases of associated CSF leakage and infection had been reported.

## Discussion

Routine surveillance at Rhode Island Hospital identified a cluster of positive EVD-associated CSF cultures and infections after a transition to the Medtronic Duet EVD system. An investigation of cases identified at Rhode Island Hospital identified frequent reports by staff members of disconnections and breaks in the Medtronic Duet EVD system tubing, and numerous reports of similar events were identified in the FDA MAUDE database. Based on these findings and the known risk for infectious complications associated with unintentional disconnections and breaks in EVD systems, investigators hypothesized that the increased number of positive CSF cultures and confirmed infections observed at Rhode Island Hospital were related to disconnections and breaks in Medtronic Duet EVD systems. Based on the potential for catheter disconnection from the patient-line stopcock connectors and reports of CSF leakage and infection, the Medtronic Duet EVDs was recalled by Medtronic Neurosurgery in January 2024 and classified as a Class I recall, the most serious type of recall, by the FDA. These findings have national implications, because Medtronic is among the largest suppliers of EVD systems in the United States, and the Duet EVD system is frequently used in hospitals across North America.

This investigation highlights the importance of both hospital infection surveillance programs and national reporting databases, such as the MAUDE database, for identifying and quickly responding to infectious outbreaks. Health care institutions in the United States are required to perform surveillance for numerous infections; however, surveillance for EVD-associated infections is not mandated by U.S. regulatory agencies. Given the potential for EVD-associated infections to result in prolonged intensive care unit and hospital length of stay, increased morbidity, and increased health care costs, this is an area for further exploration by hospital infection prevention and control programs.

### Limitations

The findings in this report are subject to at least three limitations. First, this investigation was conducted at a single center and consisted of a short follow-up period of approximately 6 weeks. Despite this limitation and the relatively small number of events observed, this analysis identified a statistically significant increase in positive CSF cultures and confirmed EVD infections after replacement of Integra Life Sciences EVD systems with Medtronic Duet EVD systems in mid-September 2023. The numerous reports of disconnections and breaks in components of the Medtronic Duet EVD in the FDA MAUDE database and the decision to recall the Duet EVD system product indicate that the issues identified in this investigation were not limited to Rhode Island Hospital. Second, although this report offers data from an investigation at Rhode Island Hospital and from the MAUDE database suggesting an association between the Medtronic Duet EVD system and a resulting increase in infectious complications, these findings do not definitively prove that the observed increase in positive CSF cultures and confirmed infections were caused by malfunctions of the Medtronic Duet EVDs that resulted in disconnections and breaks of its components. Finally, CSF samples are only collected at the discretion of the NCCU and neurosurgery teams. This limitation might have led to an underestimation of the number of patients affected by issues with the Medtronic Duet product and an underestimation of patients with positive CSF cultures because not every patient who sustained an EVD disconnection or break event would have become clinically ill or had CSF cultures collected.

### Implications for Public Health Practice

This investigation highlights the importance of hospital infection prevention and control programs in effective identification and response to clusters or outbreaks of health care–associated infections. Conducting surveillance for EVD-associated infections is not currently mandated by U.S. regulatory agencies. However, given the widespread use of EVD systems by neurosurgery centers and the risk for EVD-associated infections, a strategy for future consideration by hospital infection prevention and control programs might be inclusion of EVD-associated infections in hospital surveillance programs to rapidly identify and determine factors related to such infections to prevent additional infections.

## Footnotes

* Rhode Island Hospital is managed by Lifespan Health System, and Lifespan’s Institutional Review Board (IRB) has determined that outbreak investigations are critically time sensitive and crucial for direct patient care. Infectious disease outbreak investigations at Rhode Island Hospital fall under the purview and authority of infection control, and IRB human subjects review is not required.

† For the purposes of surveillance for infections resulting from EVD placement at Rhode Island Hospital, CSF cultures that were positive at the time of EVD insertion, within 48 hours of EVD insertion, or were positive but attributable to another source of infection, were not included in this analysis.

§ The RR for infectious events observed (positive CSF cultures and confirmed infections meeting NHSN criteria) was based on the total device days of observation using the exact Poisson method. The p-values were obtained using the chi-square statistic.

¶ The initial search results from the MAUDE database were screened, and non-Medtronic Duet devices and entries were eliminated from the analysis.
